# Rescue of Methyl-CpG Binding Protein 2 Dysfunction-induced Defects in Newborn Neurons by Pentobarbital

**DOI:** 10.1007/s13311-015-0343-0

**Published:** 2015-03-10

**Authors:** Dongliang Ma, Su-In Yoon, Chih-Hao Yang, Guillaume Marcy, Na Zhao, Wan-Ying Leong, Vinu Ganapathy, Ju Han, Antonius M. J. Van Dongen, Kuei-Sen Hsu, Guo-Li Ming, George J. Augustine, Eyleen L. K. Goh

**Affiliations:** 1 Programme in Neuroscience and Behavioral Disorders, Duke-NUS Graduate Medical School, Singapore, Singapore; 2 Lee Kong Chian School of Medicine, Nanyang Technological University, Singapore, Singapore; 3 Department of Pharmacology, College of Medicine, National Cheng Kung University, Tainan, Taiwan; 4 Department of Forensic Medicine, Key Laboratory of Health Ministry for Forensic Science, Xi’an Jiaotong University School of Medicine, Xi’an, Shaanxi People’s Republic of China; 5 Department of Physiology, Yong Loo Lin School of Medicine, National University of Singapore, Singapore, Singapore; 6 Institute for Cell Engineering, Johns Hopkins University School of Medicine, Baltimore, MD USA; 7 Center for Functional Connectomics, Korea Institute of Science and Technology, Seoul, Republic of Korea; 8 Institute of Molecular and Cell Biology, A*STAR, Proteos, Singapore, Singapore; 9 KK Research Center, KK Women’s and Children’s Hospital, Singapore, Singapore

**Keywords:** Rett syndrome, newborn neurons, dendrites, GABA, pentobarbital

## Abstract

Rett syndrome is a neurodevelopmental disorder that usually arises from mutations or deletions in methyl-CpG binding protein 2 (MeCP2), a transcriptional regulator that affects neuronal development and maturation without causing cell loss. Here, we show that silencing of MeCP2 decreased neurite arborization and synaptogenesis in cultured hippocampal neurons from rat fetal brains. These structural defects were associated with alterations in synaptic transmission and neural network activity. Similar retardation of dendritic growth was also observed in MeCP2-deficient newborn granule cells in the dentate gyrus of adult mouse brains *in vivo*, demonstrating direct and cell-autonomous effects on individual neurons. These defects, caused by MeCP2 deficiency, were reversed by treatment with the US Food and Drug Administration-approved drug, pentobarbital, *in vitro* and *in vivo*, possibly caused by modulation of γ-aminobutyric acid signaling. The results indicate that drugs modulating γ-aminobutyric acid signaling are potential therapeutics for Rett syndrome.

**Electronic supplementary material:**

The online version of this article (doi:10.1007/s13311-015-0343-0) contains supplementary material, which is available to authorized users.

## Introduction

Rett syndrome (RTT) is an X-linked dominant progressive neurodevelopmental disorder that primarily affects females and is sporadic in most cases, with a worldwide incidence of 1 in 10,000–15,000 female births [1]. Girls born with this syndrome develop normally until 6–18 months of age and then fail to achieve, or regress from, the expected developmental growth milestones. They also lose motor skills and speech, and display stereotypic hand-wringing motions. Most patients also develop other symptoms such as breathing abnormalities, hypoactivity, ataxia, seizures, and scoliosis, followed by cognitive deterioration [2]. Spontaneous mutations in the gene encoding the methyl-CpG-binding protein 2 (MeCP2) located at Xq28, have been identified as the underlying cause of most cases of RTT [1]. As the name suggests, MeCP2 binds preferentially to DNA on methylated CpG and recruits other co-repressors, such as SIN3 transcription regulator family member A (Sin3A) histone deacetylases or nuclear receptor co-repressor 2/SMRT co-repressor complexes, to influence transcription [3, 4]. MeCP2 is responsible for stable and reversible repression of downstream methylated targets, although emerging evidence suggests that MeCP2 may also switch on certain genes and regulate RNA splicing [5]. Thus, a loss of MeCP2 would lead to inappropriate gene expression.

As the phenotypes of RTT manifest themselves over specific stages and develop gradually during childhood, MeCP2 deficiency has been most prominently linked to deficiencies in postnatal neuronal development. However, a study demonstrating effects of knocking out MeCP2 in adult animals indicates that MeCP2 is also required for adult neural function [6]. The progression and pathology of RTT and MeCP2-related disorders are still poorly understood. Phenotypic reversal of MeCP2 deficiency was achieved by restoring MeCP2 function in a RTT syndrome mouse model (MeCP2 knockout) [7], as well by systemic administration of an adeno-associated virus bearing MeCP2 cDNA [8], suggesting that RTT may be potentially treatable in humans. However, gene therapy for neurologic ailments in human patients has been difficult to achieve. Therefore, it is desirable to identify drugs that can overcome the defects associated with RTT. Aminoglycoside antibiotics, such as gentamicin, have been shown to cause a partial read-through of nonsense mutations within *MECP2* [9–12]. Gentamicin has also been shown to increase MeCP2 protein levels and synapse numbers in neurons differentiated from induced pluripotent stem cells derived from patients with RTT [13]. Growth factors such as insulin-like growth factor-1 can also partially reverse RTT-like symptoms and phenotypes in MeCP2 mutant mice and neurons differentiated from RTT induced pluripotent stem cells [13, 14], while acute benzodiazepine treatment can transiently abolish the breathing defects of MeCP2-deficient mice and alter expression of brain-derived neurotrophic factor in mouse hippocampus [15, 16]. However, none of these approaches causes complete recovery of normal function, so that identification of better therapeutic agents for RTT is necessary.

It has been shown that MeCP2-related disorders display neurologic phenotypes that can be attributed, in part, to postnatal hippocampal dysfunction [17–19]. We have therefore examined the cell-autonomous actions and functional role of MeCP2 in the development and maturation of newborn cells from the hippocampus of fetal brains, and also in newborn cells in the adult hippocampus *in vivo*. MeCP2 deficiency resulted in changes in dendritic structure, reduced synapse number and impaired synaptic transmission and reduced neuronal network activity. We also demonstrate that pentobarbital (PB) can reverse the structural and synaptic phenotypes resulting from MeCP2 deficiency, revealing the potential of γ-aminobutyric acid (GABA) A receptor (GABA_A_R) modulators such as PB as novel therapeutic treatments for RTT in the future

## Materials and Methods

### Construction, Production, and Stereotaxic Injection of Engineered Retroviruses

High titers of engineered self-inactivating murine retroviruses or lentiviruses (1 × 10^9^ unit/ml) were produced as previously described [20, 21]. Two short hairpin RNAs (shRNAs) targeting different regions of mouse MeCP2 and one control shRNA with a scrambled sequence were designed and cloned into retroviral or lentiviral vectors: GGGAAACTTGTTGTCAAGATGCC (sh834 or shMeCP2); GGAGTCTTCCATACGGTCT (sh1049); AGTTCCAGTACGGCTCCAA (shctrl). A shRNA with a mutated sh834 (GCGAAACTCGTTGTTAAGATGGC) sequence was generated (shMut) to further validate the specific effects of MeCP2 knockdown in mice. GGGAAACTTCTCGTCAAGA (shMeCP2 against rat) was cloned into a lentiviral construct for knocking down MeCP2 in rat primary hippocampal neurons. Lentiviral expression constructs of mouse MeCP2–internal ribosomal entry site–enhanced green fluorescent protein were co-transfected with retroviral constructs carrying shctrl, sh834, shMut (shRNA with mutated sh834), or sh1049 into human embryonic kidney (HEK) 293 cells to validate the specificity and efficiency of the shRNAs using Western blot analysis. Lentivirus carrying either shctrl or sh834 (henceforth termed “shMeCP2”) were produced to infect postmitotic primary hippocampal neurons to confirm the specificity and efficiency of the shRNAs in these neurons using Western blot analysis [at 14 days *in vitro* (DIV)] and immunofluorescence (at 5 DIV).

**Fig. 1:**
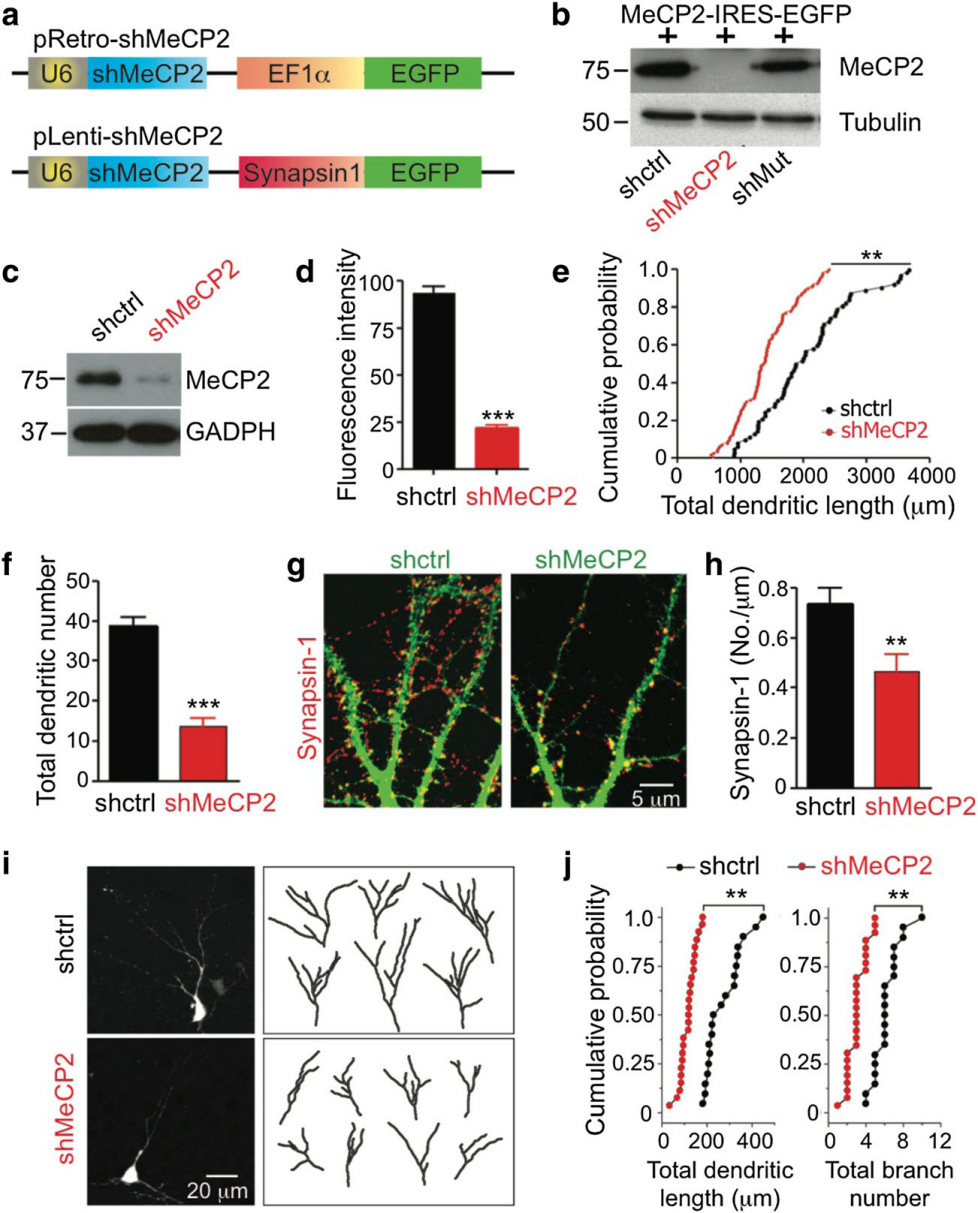
Methyl-CpG-binding protein 2 (MeCP2) deficiency affects dendritic development and synaptogenesis in hippocampal neurons. (a) Schematic diagram showing the knockdown retroviral and lentiviral constructs. EF1α = elongation factor 1- α; EGFP = enhanced green fluorescent protein. (b) Representative Western blot showing the efficiency of retrovirus carrying short hairpin RNA (shRNA) of scrambled sequence or shRNAs against MeCP2 (shMeCP2, shMut) in knocking down MeCP2 in human embryonic kidney 293 cells overexpressing MeCP2 using Western blot analysis at 5 days *in vitro* (DIV). IRES = internal ribosomal entry site. (c) Representative Western blot showing the efficiency of lentivirus with shMeCP2 in knocking down MeCP2 in embryonic day 18 (E18) primary hippocampal neurons at 14 DIV. GAPDH = glyceraldehyde 3-phosphate dehydrogenase. (d) Average fluorescence intensity per E18 hippocampal neurons (at 5 DIV) infected with lentivirus carrying scrambled RNA (shctrl) or shMeCP2 (***p < 0.01; Student’s *t* test) (e) Quantification of total dendritic length and (f) total dendritic branch number of shctrl- or shMeCP2-expressing cells (***p < 0.01; Student’s *t* test). (g) Representative images of neurons expressing shctrl or shMeCP2 (green), immunostained with anti-synapsin I (red) (scale bar = 5 μm). (h) Density of synapsin-1 positive puncta per μm dendrite in hippocampal neurons transfected with shctrl or shMeCP2 (**p < 0.01; Student’s *t* test). (i) Representative images and tracings of dendritic arbors of dentate gyrus granule neurons infected with retrovirus carrying shctrl (upper panel) or shMeCP2 (lower panel) *in vivo* (scale bar = 5 μm). (j) Quantitative analysis of the (left) total dendritic length, (right) total branch number of control, and MeCP2 deficient neurons (**p < 0.01; Student’s *t* test)

For *in vivo* studies, adult (7–8 weeks old) female C57Bl/6 mice were anesthetized and different sets of retroviruses were stereotaxically injected into the dentate gyrus at 4 sites. The mice were euthanized at 14 days after viral injection for morphological analysis, as previously described [20, 21]. All animal procedures and applicable regulations of animal welfare were in accordance with institutional animal care and use committee guidelines. For pharmacologic modulation *in vivo*, mice received intraperitoneal injections of PB (1 mg in 100 μl saline per mice) every 2 days after virus injection, until day 14, when they were euthanized for analysis.

### Hippocampal Neuron Primary Cultures

Primary cultures of hippocampal neurons were isolated from embryonic day 18 (E18) Long–Evans rats as described previously [21–23]. Briefly, the hippocampus was carefully extracted from the brain and collected in buffer (127 mM NaCl, 5 mM KCl, 170 μM Na_2_HPO4, 205 μM KH_2_PO4, 5 mM glucose, 59 mM sucrose, 100 U/ml penicillin/streptomycin, pH 7.4). Cells were dissociated, washed, and collected in growth medium [Dulbecco’s Modified Eagle’s Medium w/GlutaMax (Invitrogen, Carlsbad, CA, USA) containing 1 M 4-(-hydroxyethyl)-1-piperazineethanesulfonic acid (HEPES), 10% heat inactivated horse serum (Invitrogen), and 100 U/ml penicillin/streptomycin (pH 7.4)]. Cells were plated at 1.5 × 10^6^ cells per 24-well plate for all *in vitro* experiments, including morphological analysis, calcium imaging, and electrophysiological recordings. Cytosine arabinoside (10 μM) was added to the neuronal culture to eliminate dividing cells (astrocytes and microglia). Neuronal cultures were infected with a lentiviral vector carrying shctrl or shMeCP2, and cultured for 12–14 days. For chronic drug treatment, neurons were infected with virus and then grown for another 24 h before being treated with vehicle, 5 μM PB, 5 μM bicuculline, or 25 μM musimol. Culture media were replaced with fresh media with drugs every other day throughout the entire culture period. At least 5–6 batches of culture were used for each experiment. Each batch of cultures was isolated from pooled hippocampus of all E18 pups (typically 8–10) from 1 animal.

### Electrophysiology

Whole-cell patch clamp recordings were performed on primary hippocampal neurons (12–14 DIV) at room temperature in an external solution containing 127 mM NaCl, 2.6 mM KCl, 23.8 mM NaHCO_3_, 0.77 mM NaH_2_PO_4_, 2 mM MgCl_2_, 2.5 mM CaCl_2_, and 10 mM glucose aerated continuously with 95% O_2_/5% CO_2_. Recording pipette resistance ranged from 4 to 6 MΩ. Series resistance ranged from 10 to 20 MΩ and was monitored throughout the recordings. The recordings were made at a holding potential of -70mV using a Multiclamp 700B amplifier (Molecular Devices, Sunnyvale, CA, USA), filtered at 2 kHz and digitized at 10 kHz with a Digidata 1440A (Molecular Devices).

Miniature excitatory postsynaptic currents (mEPSCs) were recorded in the presence of 1 μM tetrodotoxin (TTX) and 20 μM bicuculline. For such recordings, the low chloride internal solution contained 120 mM K-gluconate, 9 mM KCl, 10 mM KOH, 4 mM NaCl, 10 mM HEPES, 1 mM ethylene glycol tetraacetic acid, 2 mM Mg_2_ATP, and 0.4 mM Na_3_GTP (pH 7.4, 295 mOsm). mEPSCs were identified and isolated using Minianalysis (Synaptosoft, Seoul, Korea). These events were then manually inspected to discard any events that did not represent mEPSCs.

Because the cultures consisted of both excitatory and inhibitory neurons, we took the following measures to ensure that differential contributions from neuronal subtypes did not bias the data: 1) neurons were randomly selected; 2) data were recorded from a large number of neurons (in all cases at least 10 cells, as indicated in the figure legends); 3) blind experiments were performed so that the person doing the recordings did not know which experimental group the neurons came from.

### Calcium Imaging

Hippocampal primary neurons were washed twice with loading buffer containing the following: 118 mM NaCl, 4.69 mM KCl, 4.2 mM NaHCO_3_, 1.18 mM KH_2_PO, 0.8 mM MgCl_2_, 2.0 mM CaCl_2_, 20 mM HEPES, and 30 mM glucose (pH 7.4), and incubated with final concentration of 1 μM X-rhod-1 (Molecular Probes/Invitrogen, Carlsbad, CA, USA) for 30 min at 37°C. Excess dye was removed by washing twice with loading buffer, and an additional 20-min incubation was done to equilibrate the intracellular dye concentration and allow for de-esterification. Time-lapse image sequences of 500 frames were acquired with a sampling rate of 0.64 Hz with a region of 512 × 512 pixels, with 488-nm (fluorescein isothiocyanate) and 534-nm filters on a LSM 710 inverted fluorescence confocal microscope (Carl Zeiss, Singapore). Images were acquired with ZEN software (Carl Zeiss) and data analysis was done using MATLAB (Mathworks, Natick, MA, USA). At least 20 green fluorescent protein-positive neurons were randomly selected for calcium imaging (as indicated in the figure legends) from at least 3 coverslips/batch.

For each experiment, 20 regions of interests were selected to record the calcium intensity of 20 cells under fluorescence, with a sampling rate of 1.56 s/frame. The typical firing rate of these cells in these experiments is 2–20 spikes/min [24], and the duration of each calcium spike is normally longer than 2 s; therefore, our sampling rate was high enough to capture spike events. Peak detection was done in MATLAB according to previous studies [13], with the criterion of ∆F/F > 10%, where F represents calcium intensity and ∆F is the change of intensity in consecutive frames. The amplitude of each peak is measured by the difference between the peak value and the baseline.

The correlation coefficient r_xy_ (τ) for each pair of neurons was computed based on previously published equations [25]. A peak at the center, where τ = 0, means the 2 neurons are synchronized, while a shifted peak indicates a likely causal relationship with certain delay. For each culture, the average correlation coefficient of all possible pairs among the 20 selected neurons was calculated. Neurons that did not generate detectable spikes were excluded from the calculation. To measure the strength of synchronicity in each culture, a synchronicity index was obtained by the average difference between the peak in the correlation coefficient and the baseline value [26, 27]. Therefore, a higher synchronicity index indicates stronger synchronization in activity. The cells that did not have detectable spikes were excluded when calculating average peak amplitudes of calcium spikes. For interspike interval statistics, only the cells that had > 2 spikes were taken into consideration.

### Confocal Imaging and Analysis

Cells were fixed and processed for the immunocytochemistry procedure using antibodies against Synapsin-1 (1:500; Abcam, Cambridge, UK), together with 4’,6-diaminodino-2-phenylindole (1:5000). Images were acquired on a LSM 710 confocal microscope or LSM 7 ELYRA PS.1 system (Carl Zeiss, Singapore). Coronal brain sections (40 μm) were prepared from virus-injected mice and processed as previously described [20, 21]. The projected 3-dimensional reconstruction images were semiautomatically traced, and total dendritic length and branch number of each individual GFP-positive neuron in the granule cell layer were analyzed. The distribution patterns of dendritic arborization of each individual neuron under different conditions are shown in accumulative distribution plots. Statistical significance (*p* < 0.01) was assessed using the Kolmogorov–Smirnov test. Sholl analysis for dendritic complexity was carried out by counting the number of traced dendrites that cross a series of concentric circles at 5-μm intervals from the cell soma. Statistical significance (*p* < 0.05) was assessed using the Student’s *t* test.

## Results

### MeCP2 Deficiency Affects Neuronal Development

To examine the role of MeCP2 in neuronal development, we used shRNA to silence MeCP2 expression in HEK293 cells via retrovirus or in primary hippocampal neurons from rat fetal hippocampus by using lentivirus (Fig. 1a–d). The efficiency of knockdown was determined by Western blot analysis and immunocytochemistry (Fig. 1b–d). We found that a specific MeCP2-directed shRNA (shMeCP2) was very effective in reducing MeCP2 levels, while a scrambled construct (shctrl) did not (Fig. 1b–d). As a further control, we found that a point-mutated version of the active shRNA (shMut) was also ineffective in reducing MeCP2 levels (Fig. 1b).

Silencing MeCP2 in cultured neurons resulted in less elaborate outgrowth of neurites compared with cells infected with the scrambled control, shctrl (Fig. 1e,f). This was evident both as a reduction in the total length of dendrites and as a decrease in the number of dendrites (Fig. 1e,f). We also observed a significant reduction in the number of synapses per dendritic trunk (length in μm), as detected with immunostaining for synapsin-1 (Fig. 1g, h), a marker of presynaptic terminals.

Next, to examine the cell-autonomous effect of MeCP2 *in vivo*, we injected retrovirus carrying shRNA directed against MeCP2 *in vivo* into the dentate gyrus of adult mouse brains, allowing genetic manipulation of individual progenitor cells in the adult dentate gyrus. This resulted in less elaborate dendritic arborization than in control cells that received the control shRNA (Fig. 1i, j), indicating a direct and cell-autonomous effect of MeCP2 on these cells. This was evident in measurements of the total length of dendritic processes and the number of dendritic branches (Fig. 1j, left and Fig. 1j, right, respectively). These results demonstrate that loss of MeCP2 has similar effects in dendrite development, both on cultured newborn hippocampal neurons *in vitro* from early development and on newborn neurons *in vivo* in developed adult brains.

### MeCP2 Deficiency Affects Functional Properties of Neurons

**Fig. 2:**
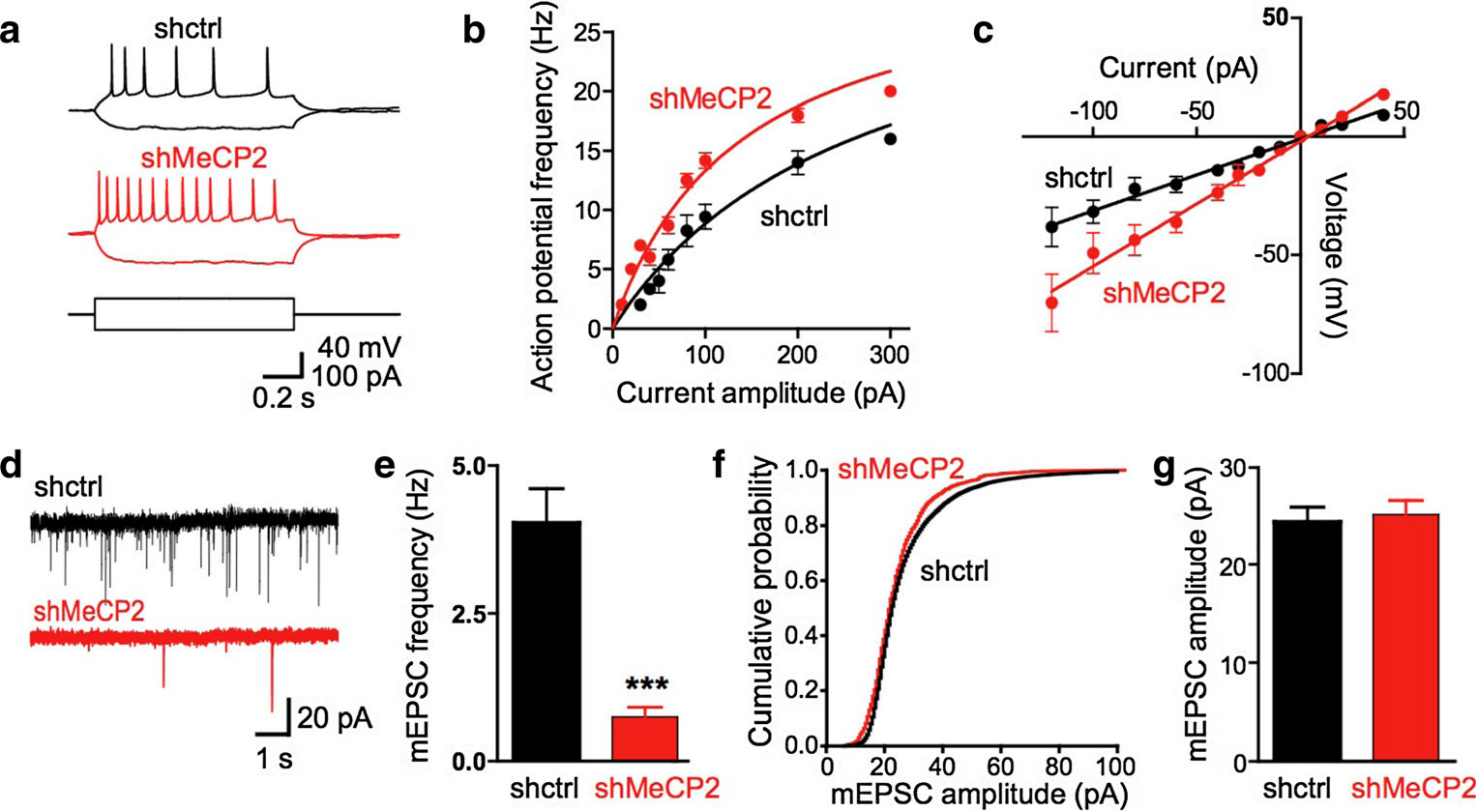
Methyl-CpG-binding protein 2 (MeCP2) deficiency affects functional properties of neurons. (a) Representative traces and (b) quantitative analysis of action potentials evoked in response to depolarizing current pulses in cultured hippocampal neurons expressing scrambled RNA (shctrl) or short hairpin RNA against MeCP2 (shMeCP2). (c) Relationship between amplitude of injected current pulses (Current; 1 s duration) and size of resulting change in membrane potential (Voltage) in cultured hippocampal neurons expressing shctrl or shMeCP2. (d) Representative traces of miniature excitatory postsynaptic currents (mEPSC) recorded in the presence of 1 μM tetrodotoxin and 20 μM bicuculline. (e–g) Quantitative analysis of mEPSCs showing the frequency and amplitude of mEPSC in the control and MeCP2 knockdown cells. The number of cells analyzed was > 10 per condition. ***p < 0.01 (Student’s *t* test)

To determine whether the dendrite defects caused by MeCP2 knockdown are translated into functional defects, we next examined the effects of MeCP2 knockdown on the functional properties of neurons. MeCP2 knockdown caused substantial changes in the intrinsic properties of cultured neurons. We found that the membrane capacitance (C_m_) was smaller in MeCP2 knockdown neurons than in control cells (Table 1). Because C_m_ is proportional to the plasma membrane surface area, this reduction in C_m_ is presumably due to the shorter dendritic length of these cells (Fig. 1e,f). Likewise, the input resistance of MeCP2-deficient neurons was higher than that of control cells (Table 1). This is also consistent with the smaller plasma membrane area of these neurons because input resistance varies inversely with surface area. While the resting membrane potential of MeCP2 knockdown neurons was similar to that of control neurons, the number of action potentials evoked in response to depolarizing current pulses was higher (Fig. 2a,b). This presumably arises from the higher input resistance of these neurons, which creates larger voltage changes in response to a given amount of injected current. This can be seen in the current–voltage relationships shown in Fig. 2c; the slopes of these curves (the membrane resistance) were significantly different (t-stat = 9.149, *p* < 0.01) for control and MeCP2 knockdown neurons. A larger depolarization will generate more action potentials, as seen in Fig. 2a, b. There were no significant differences (*p* = 0.95) in action potential threshold or amplitude between control and knockdown neurons (Table 1). There was a modest (10%) reduction in spike duration, which might make a minor contribution to the increase in action potential firing. Thus, loss of MeCP2 does not significantly affect the intrinsic excitability of these cells but simply makes them smaller. This indicates that MeCP2 plays an indirect role in regulating neuronal excitability via changes in surface area.

**Table 1:** Electrophysiological properties of cells transfected with either scrambled RNA (shctrl) or short hairpin RNA against MeCP2 (shMeCP2)

	Capacitance (pF)*	Input resistance (MΩ)*	Resting membrane potential (mV)^†^	Spike voltage threshold (mV)^‡^	Spike amplitude (mV)^‡,§^	Spike width (ms)^‡,¶^
shctrl	94.1 ± 3.1	255.7 ± 16.5	–72.4 ± 2.1	–49.4 ±1.1	74.6 ± 1.3	2.8 ± 0.5
shMeCP2	72.8 ± 3.2	462.7 ± 51.0	–70.4 ± 0.3	–49.4 ± 0.7	73.0 ± 2.8	2.5 ± 0.05
*p*-value (*t* test)	< 0.01	< 0.01	0.34	0.95	0.50	< 0.01

*The number of cells used to calculate mean values was 64 for shctrl and 51 for shMeCP2

^†^The number of cells used to calculate mean values was 20 for shctrl and shMeCP2

^‡^The number of cells used to calculate mean values was 8 for shctrl and 14 for shMeCP2

^§^Defined as the amplitude from threshold potential to the peak of overshoot

^¶^Measured as the width of half maximal spike amplitude

### MeCP2 Deficiency Affects Synaptic Transmission

To further examine the functional consequences caused by MeCP2 knockdown, we compared synaptic transmission in voltage clamped MeCP2 knockdown and control neurons. We first examined quantal release of the excitatory neurotransmitter, glutamate, by recording spontaneous mEPSCs (Fig. 2d). In these experiments, TTX was used to block action potential firing in presynaptic neurons, and the GABA receptor blocker bicuculline was also applied to eliminate inhibitory postsynaptic responses. The mean frequency of mEPSCs was markedly reduced in MeCP2-deficient neurons compared with control cells (Fig. 2e). Presumably, this is due to the decreased dendrite length and concomitant reduction in synaptic contacts observed in MeCP2 knockdown neurons (Fig. 1e–h). In contrast, the amplitude of mEPSCs in MeCP2-deficient neurons was comparable with controls, evident both in the distribution of mEPSC amplitudes and in the mean amplitude of mEPSCs (Fig. 2f, g). This suggests that MeCP2 knockdown affects neither presynaptic packaging of glutamate into synaptic vesicles nor the properties of postsynaptic glutamate receptors.

**Fig. 3:**
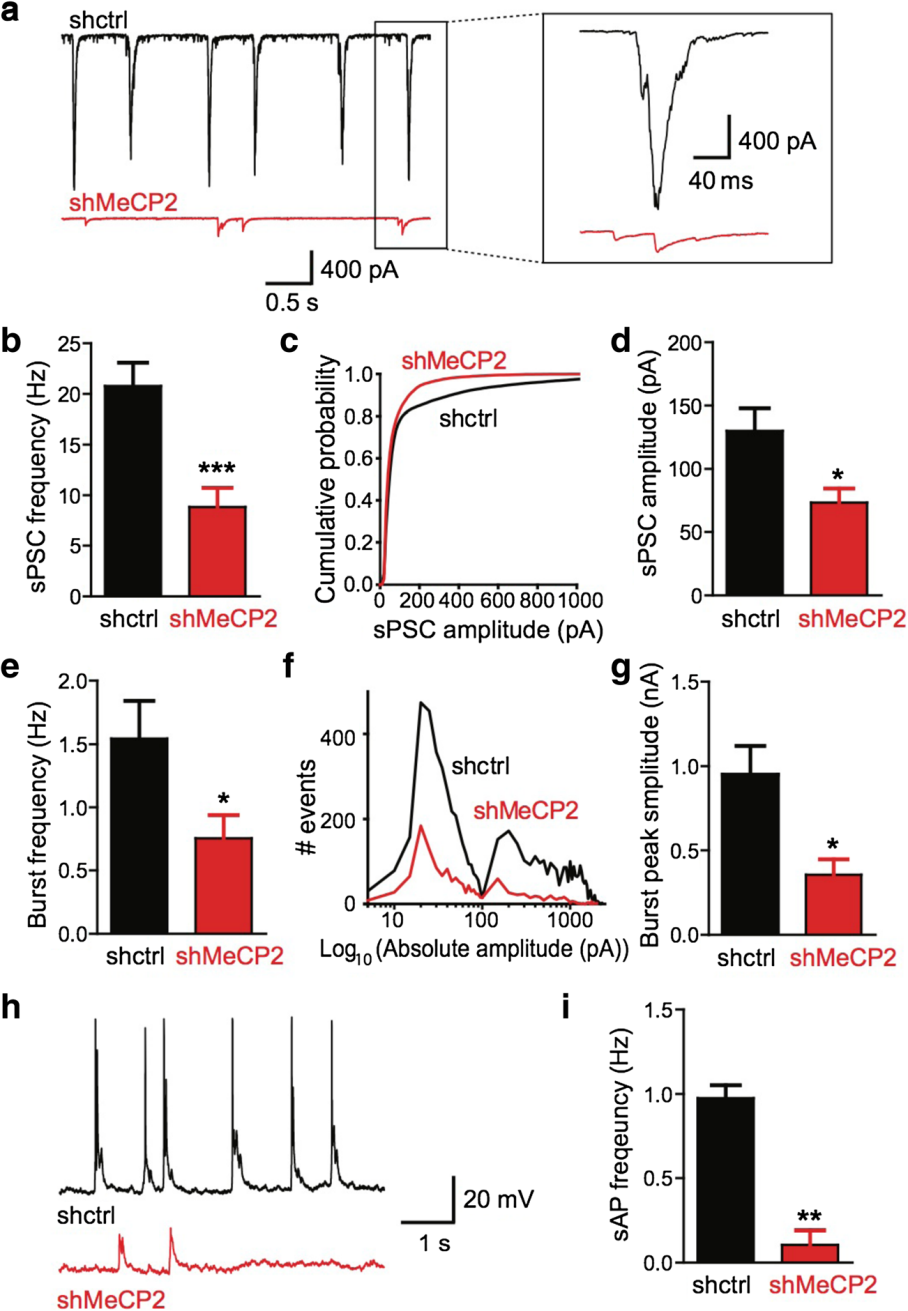
Spontaneous synaptic network excitation is reduced in response to methyl-CpG-binding protein 2 (MeCP2) knockdown. (a) Representative traces of spontaneous excitatory postsynaptic currents (EPSCs) recorded in control (upper) and MeCP2 knockdown (lower) neurons. Quantification of (b) frequency and (c, d) amplitude of spontaneous unitary synaptic currents (sPSC) in the 2 groups of cells (*p < 0.05, ***p < 0.01; Student’s *t* test). (e) Quantification of frequency of spontaneous EPSC bursts, as well as (f, g) amplitude levels achieved by EPSCs during and between these bursts in the 2 groups of cells. Note logarithmic scale in (f), to allow better visualization of both large and small EPSCs (*p < 0.05). Action potentials evoked by spontaneous synaptic excitation of (h, upper) control neurons were virtually abolished in (h, lower) MeCP2 knockdown neurons. (i) Quantification of frequency of spontaneous action potentials (sAPs) in the 2 groups of cells (**p < 0.01; Student’s *t* test)

To examine effects of MeCP2 knockdown on network synaptic activity, we recorded spontaneous postsynaptic currents in cultured hippocampal neurons in the absence of receptor blockers and without TTX (Fig. 3a). Two types of spontaneous EPSCs were observed: discrete, unitary EPSCs, and bursts of superimposed EPSCs that presumably represent synchronous excitation of the network of presynaptic neurons (see inset in Fig. 3a). Both types of EPSC were markedly reduced in MeCP2 knockdown neurons. Unitary EPSCs, including those resolvable during bursts, were present at a mean frequency of approximately 20 Hz in control neurons but their frequency was reduced by > 50% in MeCP2 knockdown cells (Fig. 3b). Analysis of the amplitudes of spontaneous EPSCs revealed a selective loss of relatively large events in response to MeCP2 knockdown (Fig. 3c), leading to a reduction in mean EPSC amplitude (Fig. 3d). Likewise, there was a reduced frequency of bursts of EPSCs in MeCP2 knockdown neurons (Fig. 3e). Analysis of peak EPSCs amplitude revealed 2 main populations of events: 1 group in the range of 10–100 pA, reflecting unitary events, and a larger group, in the range of 100–1000 pA, reflecting EPSCs in the bursts (Fig. 3f). While there were fewer events in both EPSC populations in MeCP2 KD neurons, there was a preferential reduction in the largest EPSCs within bursts (Fig. 3f). As a result, the maximum amplitude of EPSC bursts was reduced in MeCP2 knockdown neurons (Fig. 3g).

In control conditions, the spontaneous bursts of excitatory synaptic events could be observed to elicit action potentials (Fig. 3h, upper trace). Consistent with the reduction in the frequency of EPSC bursts in MeCP2 knockdown neurons (Fig. 3e), spontaneous action potentials evoked by spontaneous bursts of excitatory synaptic events were much rarer in these neurons (Fig. 3h, lower trace). Quantification revealed that the frequency of these spontaneous action potentials was greatly reduced in the MeCP2 knockdown cells (Fig. 3i). Thus, the loss of MeCP2 translates to reduced excitation within synaptic networks, despite the fact that responsiveness to depolarizing currents is increased (Fig. 2b,c).

### MeCP2 is Important for Neuronal Network Activity

**Fig. 4:**
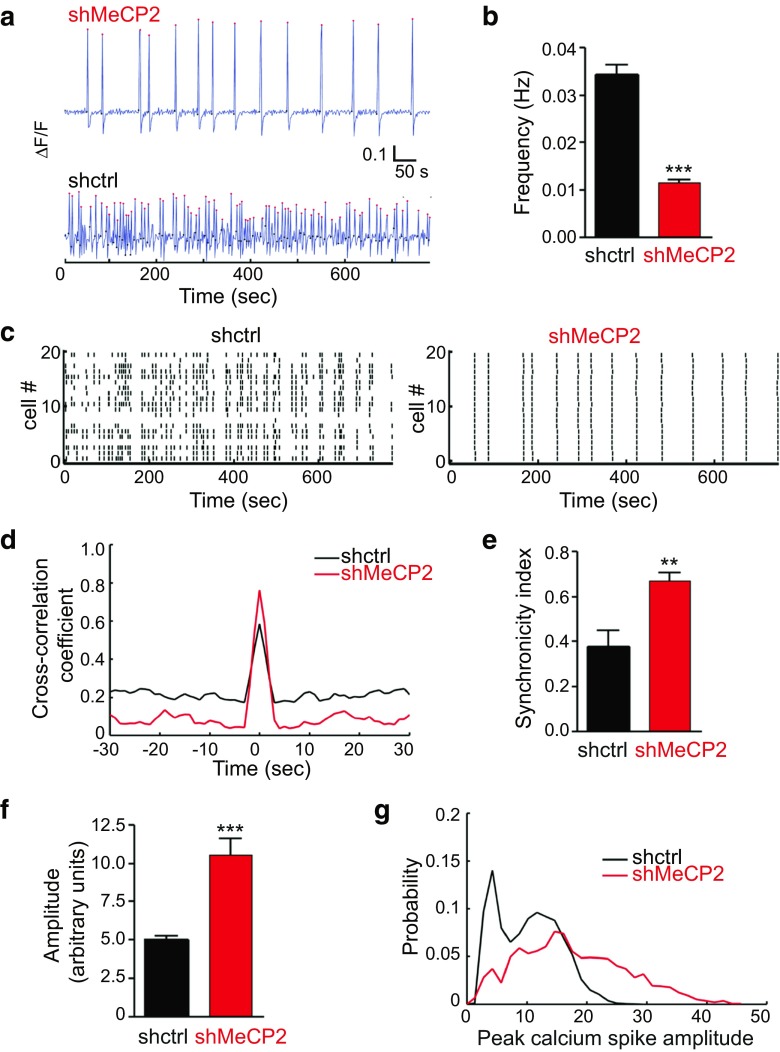
Methyl-CpG-binding protein 2 (MeCP2) is required for neuronal network activity. (a) Representative traces of spontaneous calcium spikes observed in neurons expressing either scrambled RNA (shctrl) or short hairpin RNA against MeCP2 (shMeCP2). (b) Quantitative analysis of the frequency of spontaneous calcium spikes in neurons (***p < 0.01; Student’s *t* test). (c, d) Representative spike raster plot showing calcium spikes from > 20 neurons/plot over a time period of 800 s, as indicated on the *x*-axis. (d, e) The synchronicity index calculated by taking the ratio of the coefficient index values minus the baseline value divided by the total number of cells used in the cross-correlation analysis (**p < 0.01; Student’s *t* test). (f, g) Quantitative analysis of the amplitude of spontaneous calcium spikes in neurons and the probability of distribution of peak calcium intensity (***p < 0.01; Student’s *t* test).

We next looked at the effects of these network changes on the spatial organization of neuronal activity by using calcium imaging to measure the activity of many neurons simultaneously (Fig. 4). In these hippocampal neurons, neuronal activity is associated with spontaneous and synchronous rises in intracellular calcium concentration (calcium spikes) [28]. Here, we observed a decrease in the frequency of these calcium spikes in MeCP2-deficient neurons compared with control neurons (Fig. 4a,b), presumably due to the lower rate of synaptically driven action potentials in these cells (Fig. 3h,i). Raster plots indicated that although calcium spike frequency was decreased in MeCP2-deficient neurons, these signals were much more synchronous across neurons (Fig. 4c). Cross-correlation analysis showed a higher degree of synchronization, evident as a higher peak at time = 0 (Fig. 4d). This was confirmed by calculating the synchronicity index (see “Materials and Methods”), which was significantly higher in the MeCP2-deficient neurons (Fig. 4e). In addition, there was an increase in the amplitude of calcium spikes in the MeCP2-deficient neurons (Fig. 4a, f, g). Increased synchronicity and reduced spike frequency have been suggested to result from a decrease in the number of neurons firing independently [29]. Moreover, more neurons firing synchronously leads to larger calcium spikes [29].

### PB Treatment Rescues Dendritic Defects and Synaptic Functions Associated with MeCP2 Dysfunction

**Fig. 5:**
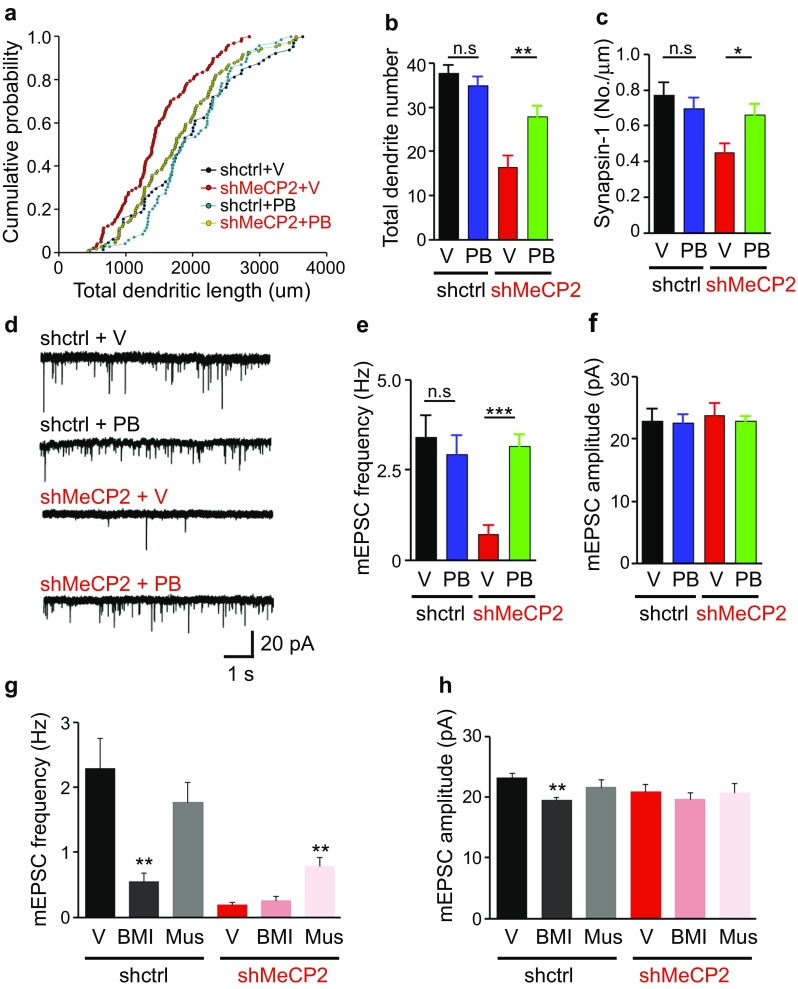
The effect of pentobarbital (PB) on reversal of cellular and functional defects caused by lost of methyl-CpG-binding protein 2 (MeCP2). Cells were grown for 24 h before treatment with vehicle or 5 μM PB. Culture media were replaced with fresh media with drugs every other day throughout the entire culture period (12–14 days *in vitro*). (a–c) Total dendritic length, branch number, and density of synapsin-1-positive puncta per μm dendrite in hippocampal neurons transfected with scrambled RNA (shctrl) or short hairpin RNA against MeCP2 (shMeCP2), treated with vehicle (V) or PB. n.s = not significant (**p < 0.01; analysis of variance–Newman–Keuls multiple comparison test). (d–f) (d) Representative traces and graphs showing quantitative analysis of (e, f) the frequency and the amplitude of miniature excitatory postsynaptic currents (mEPSCs) in hippocampal neurons transfected with shctrl or shMeCP2, treated with vehicle or PB n.s = not significant (***p < 0.01; analysis of variance–Newman–Keuls multiple comparison test). (g, h) Quantitative analysis of (g) the frequency and (h) the amplitude of mEPSCs in hippocampal neurons expressing shctrl or shMeCP2, treated with vehicle (V), 5 μM bicuculline (BMI), or 25 μM muscimol (Mus) (**p < 0.01; analysis of variance–Newman–Keuls multiple comparison test)

MeCP2 plays a role in neuronal development and connectivity (Figs. 1, 2, 3 and 4), and GABA signaling has been implicated in both [20, 30–32]. Specifically, it has previously been demonstrated that conversion of GABA-induced depolarization into hyperpolarization promotes dendrite development of newborn neurons, and GABA regulates synaptic integration of these newly generated neurons in the adult brains [20]. Moreover, the level of GABA expression in the hippocampus of MeCP2-deficient mice at p55 (late disease stage) is significantly lower than in wild-type mice [30]. Therefore, we asked whether a drug that potentiates GABA signaling can promote dendritic development of newborn neurons and thereby rescue the defects of MeCP2 dysfunction. We treated the cells with a US Food and Drug Administration-approved drug, PB, a positive allosteric modulator of GABA_A_R [33], used for the treatment of seizures and for preoperative sedation. Remarkably, compared with neurons treated with vehicle alone, chronic treatment of MeCP2-silenced neurons with PB significantly (*p* < 0.05) increased total neurite length (Fig. 5a), the number of dendritic branches (Fig. 5b), and the number of synapsin-1-positive synapses (Fig. 5c). However, PB had no significant effect on control neurons expressing scrambled control shRNA (Fig. 5a–c).

To determine whether PB could also rescue the defects in synaptic function, mEPSCs were measured in MeCP2 knockdown neurons after chronic PB treatment. In these experiments, the PB was removed immediately prior to starting the recordings in order to avoid any possible complications associated with acute effects of the PB on GABA receptors. In these conditions, the frequency of mEPSCs was significantly (*p* < 0.05) rescued by PB to values approaching those observed in control cells (Fig. 5d,e). This is consistent with the effect of PB treatment on synapse number (Fig. 5c). However, PB had no effect on mEPSC amplitude (Fig. 5d,f), which was unaffected by MeCP2 silencing. We also chronically treated the cells with an antagonist (bicuculline) and an agonist (muscimol) of GABA_A_R (Fig. 5g,h). Muscimol partially rescued the frequency of mEPSCs, while bicculline did not (Fig. 5g). This suggests that at least some of the effects of PB are due to the actions of this drug on GABA_A_R, though it is also likely that PB has additional targets.

### PB Treatment Rescues Network Activity in MeCP2-deficient Neurons

**Fig. 6:**
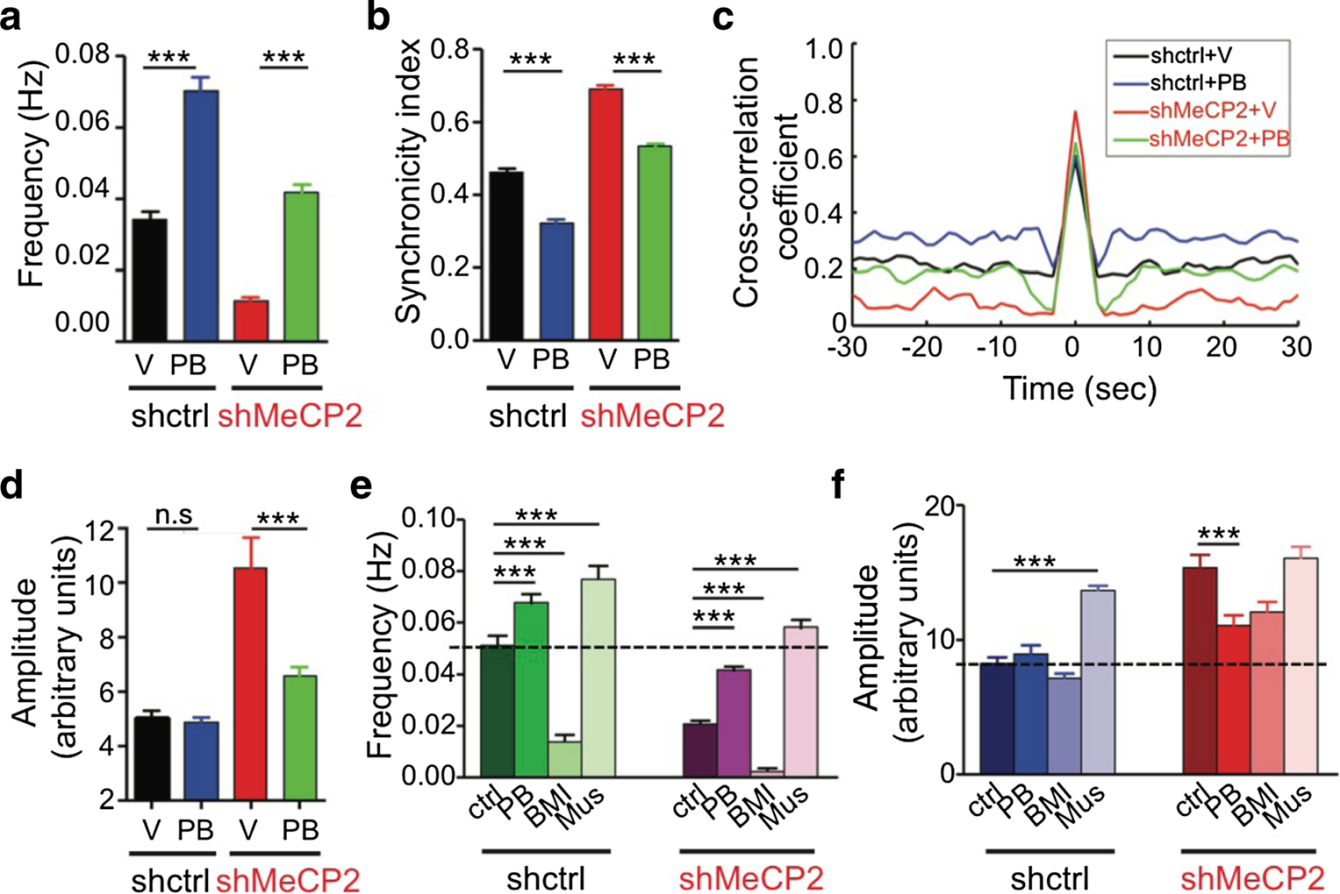
Pentobarbital (PB) treatment rescues network activity in methyl-CpG-binding protein 2 (MeCP2)-deficient neurons. (a–d) Quantitative analysis of (a) the frequency, (b, c) synchronicity, and (d) amplitude of spontaneous calcium spikes in neurons transfected with scrambled RNA (shctrl) or short hairpin RNA against MeCP2 (shMeCP2), treated with vehicle (V) or 5 μM PB. n.s = not significant. (e) Frequency and (f) amplitude of peak calcium intensity in cultured hippocampal neurons expressing shctrl or shMeCP2, treated with vehicle, 5 μM PB, 5 μM bicuculline (BMI), and 25 μM muscimol (Mus) (***p < 0.01; analysis of variance–Newman–Keuls multiple comparison test)

We next asked whether PB could reverse the effects of loss of MeCP2 on network activity. We found that PB increased the frequency of calcium spikes (Fig. 6a), and reversed the synchronizing effect of MeCP2 KD (Fig. 6b,c), although both of these effects were also observed in cells with the control shRNA (Fig. 6a–c). PB did not have any effect on the amplitude of spontaneous calcium spikes in control cells but significantly decreased the amplitude of these calcium spikes in MeCP2-deficient cells (Fig. 6d). Chronic treatment with bicuculline reduced the frequency of calcium spikes, while treatment with muscimol or PB had the opposite effect (Fig. 6e). Interestingly, bicuculline and PB, but not muscimol, reversed the increase in calcium spike amplitude observed in MeCP2 knockdown neurons (Fig. 6f). Thus, all the data are consistent with the conclusion that the functional properties of GABAergic synapses are enhanced in MeCP2-deficient cells and rescued by PB treatment.

### PB Treatment Reverses MeCP2-dependent Defects in Development of Dendrites in Newborn Neurons in the Adult Brain

**Fig. 7:**
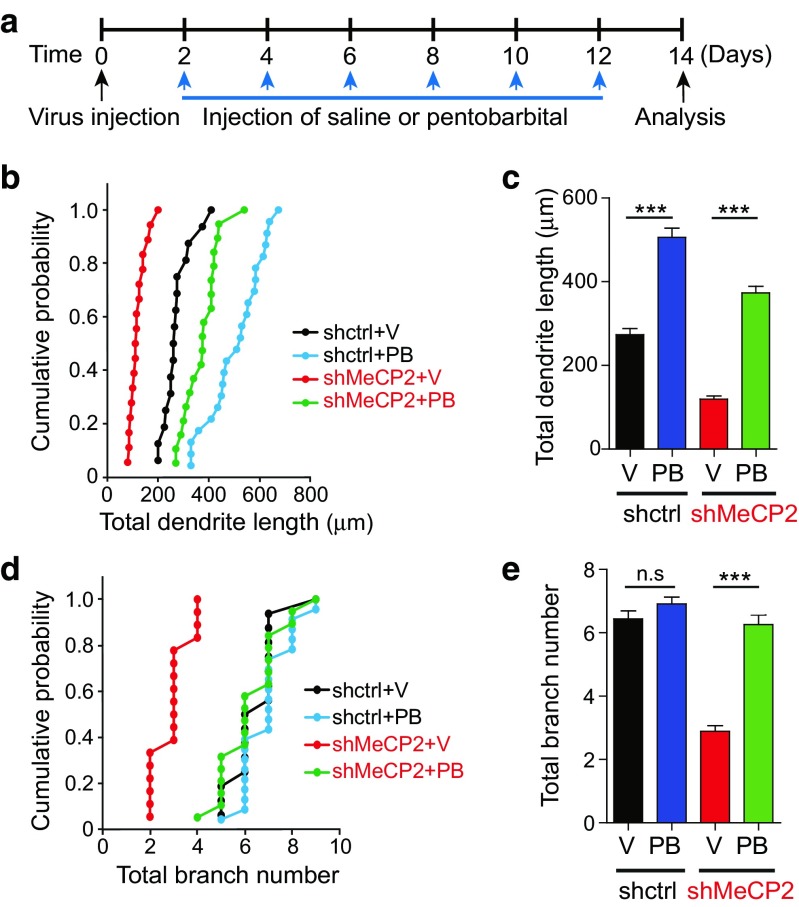
Pentobarbital (PB) rescues methyl-CpG-binding protein 2 (MeCP2)-mediated inhibition of dendritic development in newborn dentate gyrus granule cells in adult mice. Mice received intraperitoneal injections of pentobarbital (1 mg in 100 μl saline per mouse) every 2 days after virus injection, until day 14, when they were euthanized for analysis. (a) Schematic diagram showing detailed time line of the experimental procedure for chronic PB treatment. (b, c) Quantitative analysis of the total dendritic length, and (d, e) total branch number of control or MeCP2-deficient neurons treated with vehicle (V) or PB. n.s = not significant (***p < 0.01; analysis of variance–Newman–Keuls multiple comparison test)

As GABA regulates synaptic integration of newly generated neurons in the adult brains [20], we next determined whether PB, a modulator of GABA signaling, can rescue the structural defects caused by MeCP2 dysfunction *in vivo*. We analyzed neuronal morphologies after chronic PB treatment, employing the experimental design illustrated in Fig. 7a. Such chronic PB treatment increased total dendrite length and total number of branches (Fig. 7b–e). These findings suggest that PB can also compensate for the defects caused by MeCP2 dysfunction in newborn neurons *in vivo*. However, PB also enhanced dendrite outgrowth in control neurons (Fig. 7b,c). Interestingly, this morphological effect of PB on control neurons was not observed in cultured hippocampal neurons *in vitro* (Fig. 5a–c).

Taken together, our data indicate that MeCP2 knockdown causes both structural and electrophysiological phenotypes in fetal hippocampal neurons in culture. MeCP2 knockdown also causes similar, cell-autonomous defects in dendrite development in newborn neurons in the adult brain. These phenotypes can be reversed, at least in part, by chronic PB treatment both *in vitro* and *in vivo*, possibly through modulation of GABA signaling.

## Discussion

### Experimental Strategy

MeCP2 has been found to be important for dendritic branching and spine density in various types of cells, including motor cortical neurons [34], cortical pyramidal neurons [35], and hippocampal pyramidal neurons [36–38]. However, the effects on neuronal morphology vary according to age, cell type, and the particular MeCP2 mutation considered [39]. Our study focused on the effects of MeCP2 knockdown in a single brain region, the hippocampus, where MeCP2 deficiency is known to affect hippocampal functions such as learning and memory [18]. This allowed us to study the effects of MeCP2 deficiency in a well-defined population of neurons both *in vitro* and *in vivo*.

In the hippocampus of a MeCP2 knockout mouse created by deleting exon 3, containing the methyl CpG binding domain of *Mecp2*, early neurogenesis is normal but maturation is impaired, leading to defects in presynaptic protein expression and the distribution of dendritic spines [40]. This could be due to direct effects upon hippocampal neurons. However, non-neuronal cells such as glia and microglia have also been shown to play a major role in regulating dendritic morphology and synapses [41–44]. Consequently, knockout mice in which MeCP2 has been deleted from all cell types cannot distinguish between cell-autonomous and non cell-autonomous (or secondary) effects of MeCP2 in neurons. We were able to address this issue by using a retrovirus strategy to manipulate genetically individual progenitor cells in normal adult mice (Figs. 1 and 7). Our strategy took advantage of the fact that the hippocampal dentate gyrus region is one of the few brain areas in which there is continuous neurogenesis in adults. Therefore, it is an excellent system to study cell-autonomous effects of MeCP2 in individual neurons using retrovirus-mediated gene knockdown. This allowed us to circumvent the limitations associated with all currently available MeCP2 knockout mouse models of RTT. The fact that we observed dendritic abnormalities in MeCP2-deficient dentate gyrus granule newborn neurons in the hippocampus of normal adult brain indicates a cell-autonomous effect of MeCP2 deficiency in these neurons.

### Effects of MeCP2 Deficiency

We have observed that knocking down MeCP2 in cultured hippocampal neurons has several effects: it shortens dendrites, increases responses to depolarizing current pulses, reduces the number of synapses, reduces the frequency of spontaneous synaptic events, reduces the frequency of spontaneous action potentials, and reduces the number of spontaneous calcium spikes, a measure of spontaneous neuronal firing. We propose that all of these arise from a common source, namely the control of dendrite growth by MeCP2.

For a given depolarizing stimulus, shMeCP2 neurons fired action potentials at a higher frequency than control neurons (Fig. 2b). This presumably arises from the higher input resistance of these neurons, which creates a larger voltage response for a given amount of injected current, as observed in Fig. 2c. A larger depolarization will generate more action potentials, leading to the observed increased rate of action potential firing shown in Fig. 2b. The higher input resistance is consistent with, and presumably is due to, the smaller surface area of these cells (Fig. 1g). The smaller surface area can also explain the increased membrane capacitance of these cells. There were no significant differences in action potential threshold or amplitude between control and MeCP2-deficient cells. Thus, loss of MeCP2 does not significantly affect the intrinsic excitability of these cells but instead simply changes their passive electrical properties by making them smaller. There was a small (10%) reduction in spike duration, which might contribute modestly to the increased action potential firing.

The fewer synapses observed in the MeCP2 knockdown neurons is likely to be due to the less elaborate dendrites of these cells. Fewer synapses would reduce the frequency of spontaneous excitatory synaptic input, both mEPSCs and network-driven excitatory input, as observed (Figs 2e, and 3b, e). This reduction in excitatory drive should reduce spontaneous excitation of these neurons, precisely as observed (Fig. 3h, i). Further, this should decrease recurrent excitation of all neurons in the network, yielding the observed decrease in the rate of calcium spike activity.

The observed loss of synapses is in agreement with other observations demonstrating that MeCP2 regulates synaptogenesis [31, 45]. MeCP2-dependent transcriptional repression can also regulate excitatory neurotransmission in cultured hippocampal neurons. This includes studies showing that key proteins in the glutamate pathway are spatially and developmentally affected in *Mecp2-*deficient mice [30].

Spontaneous calcium spikes play a role in neural development and plasticity [13, 28], and our results showed neuronal development is affected upon knocking down MeCP2. Previous studies showed that signaling via calcium plays a substantial role in the function of MeCP2, both in regulating gene transcription and in neuronal morphology and function [36, 46]. MeCP2 has also been associated with calcium signaling, including disturbance of calcium homeostasis during early postnatal development in MeCP2 knockout mice [46]. RTT patient-derived neurons also showed altered calcium transients generated by synaptic activity [13]. The increase in the amplitude of calcium spikes in the MeCP2-deficient neurons may result from increased synchrony [29]. Similar effects of increased amplitude and decreased frequency are also seen in cortical neurons acutely treated with the GABA antagonist bicuculline [24].

### PB Rescues the Effects of MeCP2 Deficiency

The noncompetitive GABA_A_R antagonist, picrotoxin, ameliorates behavioral and synaptic impairments associated with overexpression of MeCP2 [47]. We therefore determined whether modulating GABA signaling could reverse the structural and electrophysiological phenotypes resulting from MeCP2 deficiency. We found that chronic PB treatment can reverse the morphological and functional defects caused by silencing MeCP2 in cultured primary hippocampal neurons, as well as the morphological effects observed in newly generated granule cells *in vivo*. The synaptic effects of MeCP2 loss also could be partially reversed by muscimol, a GABA receptor agonist. This suggests the involvement of GABA receptor signaling in the effects of PB.

PB is a barbiturate that binds to GABA_A_R and potentiates inhibition of GABA_A_-type receptors [33, 48]. Depending on its concentration, PB can potentiate (approximately 10–100 μM), activate (approximately 100–800 μM), or block (approximately 1–10 mM) the GABA_A_R channel [49]. The mechanisms underlying these actions remain poorly understood. Moreover, these studies were done with acute PB treatment, and the effects may differ from those caused by chronic treatment, where processes such as adaptation and tolerance of neurons may also play important roles [50]. There are only a few studies on chronic PB treatment, and these have mainly investigated its effects on expression of GABA receptor subunits. Collectively, these studies show that long-term PB treatment causes region-specific changes in the expression of GABA receptor subunits, with expression usually slightly downregulated or not significantly changed [50]. There is also little change in the maximum number of binding sites for GABA agonists such as muscimol [50]. Nonetheless, PB also has other targets in the brains, and exerts diverse effects on synaptic function apart from potentiating GABAergic transmission [48]. A better understanding of which specific components in GABA signaling is regulated and influenced by MeCP2 will be important for finding a more appropriate drug or compound that can be use as treatment for RTT. It would also be of interest to study the effects of chronic PB treatment in other brain regions in the future, in part to determine whether PB treatment could ameliorate such effects on GABA signaling.

Our data demonstrate a role for MeCP2 in growth and maturation of newborn granule cells in developing fetal and adult hippocampus, likely through GABA signaling. However, it is not clear whether MeCP2 is acting by repressing genes involved in GABA signaling or has other roles that have yet to be identified. Our results suggest that PB and related drugs that modulate GABA signaling are potential therapeutic candidates for treating RTT.

## Electronic supplementary material

Below is the link to the electronic supplementary material.

ESM 1 (PDF 1224 kb)
